# Rnf25/AO7 positively regulates wnt signaling via disrupting Nkd1-Axin inhibitory complex independent of its ubiquitin ligase activity

**DOI:** 10.18632/oncotarget.8126

**Published:** 2016-03-16

**Authors:** Rui Gao, Lin-Qiang Ma, Xiaogang Du, Ting-Ting Zhang, Liang Zhao, Luhong Liu, Jing-Crystal Liu, Fengjin Guo, Zhi Cheng, Huizhe Huang

**Affiliations:** ^1^ Second Affiliated Hospital, Chongqing Medical University, Chongqing, China 400010

**Keywords:** naked cuticle, axin, wnt, Rnf25, mesenchymal-epithelial transition

## Abstract

Wnt signaling components have been shown to control key events in embryogenesis and to maintain tissue homeostasis in the adult. Nkd1/2 and Axin1/2 protein families are required for feedback regulation of Wnt signaling. The mechanisms by which Nkd1 and Nkd2 exhibit significant differences in signal transduction remain incompletely understood. Here we report that Rnf25/AO7, a previously identified E3 ubiquitin ligase for Nkd2, physically interacts with Nkd1 and Axin in an E3 ligase-independent manner to strengthen Wnt signalling. To determine the biological role of Rnf25 *in vivo*, we found that the renal mesenchymal cell, in which *rnf25* was knocked-down, also exhibited more epithelial characters than MOCK control. Meanwhile, the transcriptional level of *rnf25* was elevated in three separate tumor tissues more than that in paracarcinomatous tissue. Depletion of Rnf25 in zebrafish embryos attenuated transcriptions of maternal and zygotic Wnt target genes. Our results indicated that Rnf25 might serve as a molecular device, controlling the different antagonizing functions against canonical Wnt signaling between Nkd1 and Nkd2 cooperated with Axin.

## INTRODUCTION

Wnt/Beta-Catenin orchestrates tissue patterning, embryonic development, adult homeostasis, and is frequently implicated in congenital malformations, cancer, and metabolic syndromes [1–3]. In vertebrates, binding of Wnt ligands to the co-receptors composed of Frizzled (Fz) and low density Lipoprotein Receptor-related Protein (LRP) 5/6 activates Dishevelled (Dvl) and promotes Axin-LRP5/6 interactions, leading to the subsequent stabilization, accumulation and nuclear translocation of Beta-Catenin, which associates with T Cell Factor (TCF)/Lymphoid Enhancer-binding Factor (LEF) family members of transcriptional factors to trigger target genes expression and thereby modulate embryonic developmental processes such as axis specification and anterior-posterior neural patterning [4, 5]. Tight regulation of Wnt/Beta-Catenin requires signal-inducible regulators to generate reproducible patterns during development, and negative feedback circuits provide efficient limiting of the signaling level and sharpening the boundaries between regions that respond differently [6]. Drosophila Naked cuticle (Nkd) is a Wg/Wnt inducible intracellular protein that antagonizes Wnt signaling in a negative feedback circuit targeting Dvl [7].

In early zebrafish development, vertebrate Nkd1 and Nkd2 both attenuate Wnt signaling but exhibit different expression patterns [8, 9]. We have noticed that the expression pattern of *nkd1* echoed the pattern of *axin2/conductin* in zebrafish embryos (Figure S1), as well as that Wnt/Beta-Catenin mediated the expressions of both *nkd1* and *axin2/conductin* [9, 10], which indicated a putative Nkd1-Axin synexpression group. Interestingly, the interaction with Axin was shown to be required for the inhibitory effect of Nkd1 on Wnt signaling, irrespective of mechanism [11, 12]. In contrast, Nkd2 is expressed ubiquitously [8] and rarely interacts with Axin (Figure S2).

Axin has emerged as a fundamental scaffold protein in multiple cell signaling pathways, including Wnt/Beta-Catenin signaling that binds to varied components in the pathway, and thus integrates inducing signals to downstream responders [13]. Distinct Axin affinity shown by Nkd1 and Nkd2 raised the intriguing possibility of the existence of novel factors engaged in the Nkd family-associated regulation of Wnt signaling. Rnf 25/AO7 is a previously established RING finger-dependent E3 ubiquitin ligase, participating in NF-kappaB [14] and EGF Receptor (EGFR) signaling [15]; and Nkd2 is one of the Rnf25 E3 targets. Here we report the identification of Rnf25 as a novel Axin-interacting protein that forms a ternary complex with Axin-Nkd1 and promotes Wnt signaling via two separate but cooperative mechanisms, which also suggests diverse roles of Nkd1 and Nkd2 in Wnt signaling.

## RESULTS

### Identification of Rnf25 as a direct Axin-interacting protein

**Figure 1:**
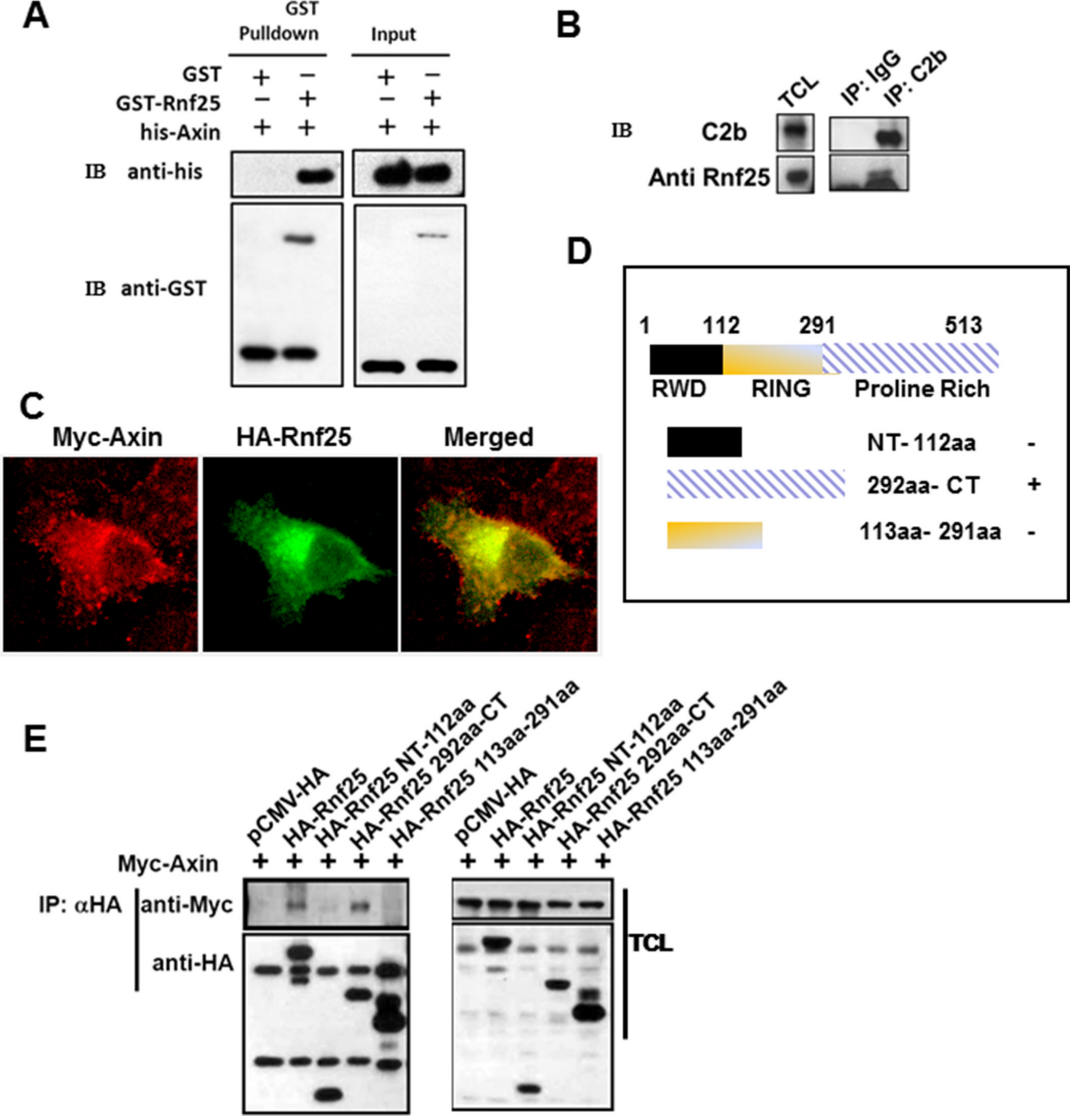
Rnf25 interacts with Axin (**A**) Purified GST-Rnf25 fusion protein pull down the RGS domain of Axin with his-taq *in vitro*. Right panel shown the input control. (**B**) Rnf25 interacts with Axin endogenously. Rnf25 is detected by anti-Rnf25 antibody in the C2b IP system. (**C**) Axin co-localizes with the Rnf25 in HeLa cells. Myc-Axin and HA-Rnf25 were co-transfected and respectively visualized by anti-Myc (Red), and anti-HA (Green). (**D**) Schematic depiction of HA-tagged wild type and deletion mutants of Rnf25. (**E**) Axin interacts with the Proline-Rich domain but not the RING domain of Rnf25. HEK293T cells were transfected with the indicated plasmids. Lanes 2 and 5 of the left panel demonstrate that Axin was precipitated when the HA-Rnf25 or HA-Rnf25 292aa-CT was expressed. IP, ImmunoPrecipitation; WB, Western Blotting; TCL, Total Cell Lysates.

Axin plays a pivotal role in Wnt signaling and is an essential factor required for integrating incoming signals by dynamic assembly of protein complexes [16]. To investigate novel Axin-interacting partners, we carried out yeast two-hybrid screen using mouse Axin RGS domain (residues 126–246) as bait and isolated three clones to encode Rnf25. To confirm the Rnf25-Axin interaction, full length of Rnf25 with GST was incubated using sepharose 4B beads and was subsequent purified from E. coli. over-expression system. The lysates containing RGS domain of Axin with his-tag was pulled-down after incubation with Rnf25 (Figure 1A). Consistent with this result, endogenous Rnf25 was co-IP with C2b antibody against Axin (Figure 1B). This interaction was further supported by the immunofluorescence assay revealing that Rnf25 was ubiquitously localized in the cytoplasm and overlapped Axin in HeLa cells (Figure 1C).

To address which domain(s) of Rnf25 mediates this interaction, three representative HA-tagged Rnf25-truncated mutants were generated, including the N-terminal fragment (residues 1–112), the middle fragment (residues 113–291) containing the RING domain responsible for its E3 ubiquitin ligase activity, and the C-terminal fragment (residues 292–513) containing the Proline-Rich domain (Figure 1D). Human embryonic kidney (HEK) 293T cells were transfected with Myc-tagged Axin, together with HA-tagged full-length Rnf25 or one of the truncated Rnf25 plasmid. Proteins in cell extracts were harvested and subjected to IP with anti-HA and WB with anti-Myc. Transfection of NT-112aa or 113aa-291aa fragment of Rnf25 did not result in immunobloting signal of Axin in the precipitate. In contrast, transfection of the Rnf25 292aa-CT fragment or of the full-length Rnf25 gave rise to a strong Axin signal in the precipitate (Figure 1E). These data suggested that Rnf25 physically interacted with Axin and the C-terminal of Rnf25 was responsible for this interaction.

### Rnf25 forms a ternary complex with axin and Nkd1

**Figure 2:**
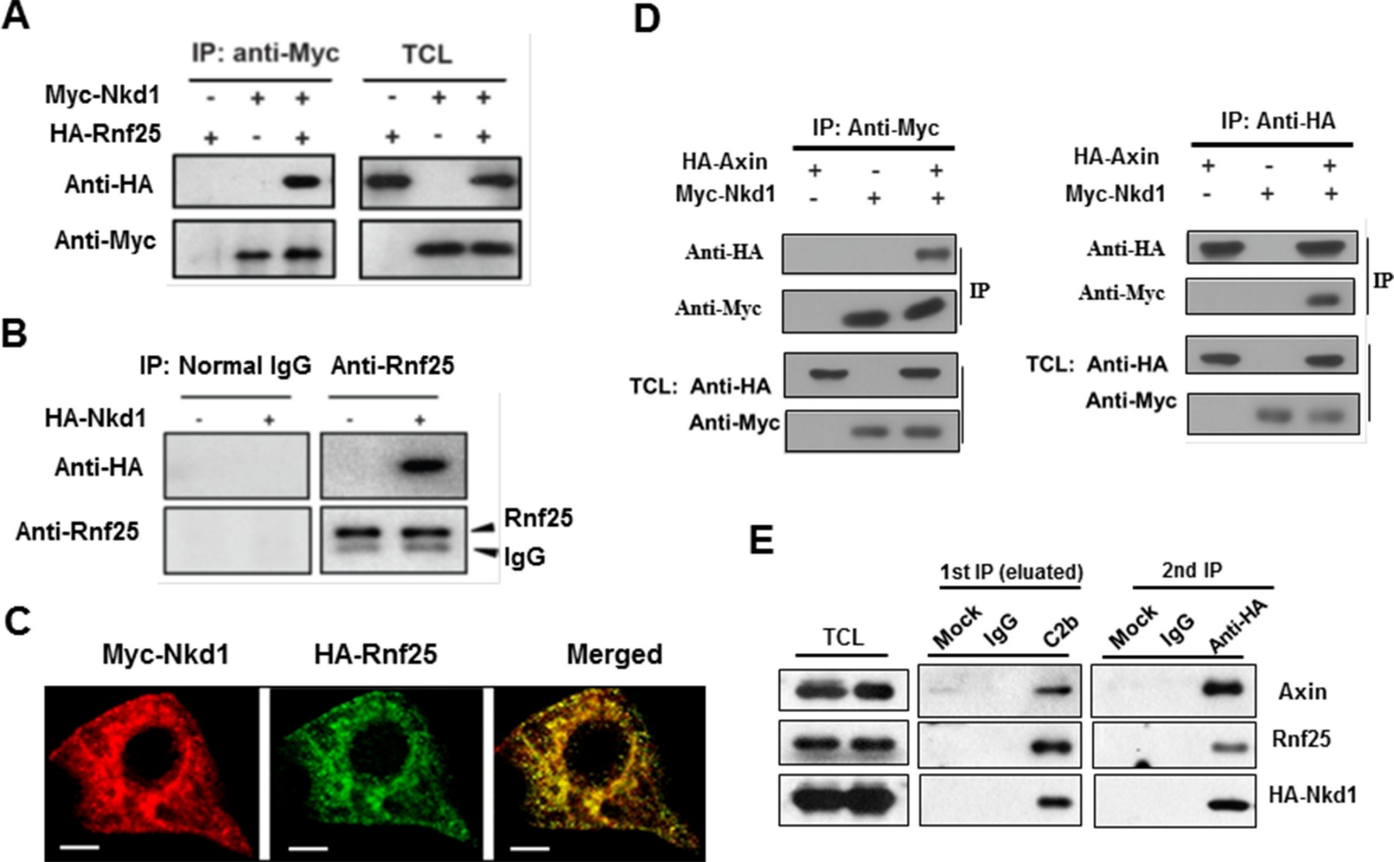
Rnf25 interacts with Nkd1-Axin complex (**A**) HA-tagged Rnf25 was expressed alone or together with Myc-tagged Nkd1 by transient transfection into HEK293T cells. Total cell lysates were immunoprecipitated with anti-Myc antibody. Protein complexes were subjected to western blotting with either anti-HA or anti-Myc antibody (left panel). Rnf25 and Nkd1 protein in total cell lysates were detected by western blot (right panel). (**B**) Endogenous Rnf25 interacts with over-expressed Nkd1. HA-tagged Nkd1 was transfected into HEK293T cells. Rnf25 was then immunoprecipitated from cell lysates using anti-Rnf25 antibody. The precipitates were detected for Nkd1 using anti-HA and for Rnf25 using anti-Rnf25 by western blotting. (**C**) Rnf25 co-localizes with the Nkd1 in HeLa cells. Myc-Nkd1 and HA-Rnf25 were co-transfected into HeLa cells. Cells were fixed and immuno-stained. Nkd1 was visualized by anti-Myc (Red), and Rnf25 by anti-HA (Green). (**D**) Nkd1 interacts with Axin. 24 h after co-transfected with HA-Axin and Myc-Nkd1 expressing plasmids, HEK293T cells were lysed and subjected to reciprocal immunoprecipitation using either anti-Myc or anti-HA antibody. Precipitations and total cell lysates were analyzed by western blotting with indicated antibodies. (**E**) Rnf25 forms ternary complex with Axin and Nkd1. 24 h post-transfected with HA-Nkd1 expressing plasmid, HEK293T cells were collected and cell lysates were subjected to two-step co-IP by firstly incubating with control IgG or C2b antibody that against to Axin. The eluate from the first immunoprecipitate was then immunoprecipitated with control IgG or anti-HA antibody followed by Western blotting using indicated antibodies.

As a well-established E3 ubiquitin ligase picking off Nkd2, homologue to Nkd that particularly attenuates Wnt signaling in an Axin-dependent manner [9], Rnf25 is recruited to Nkd2 and promoted the latter degradation via RING domain. In our overexpressed system we detected the physical interaction between Rnf25 and Nkd1 (Figure 2A). Transfection with HA-tagged Nkd1 alone followed by IP/WB assays further confirmed the binding of Nkd1 to endogenous Rnf25 (Figure 2B). Immunostaining in HeLa cells demonstrated that Nkd1 was located both in the cytoplasm and the plasma membrane and was partially co-localized with Rnf25 (Figure 2C). Reciprocal IP/WB results (Figure 2D) confirmed the direct interaction between the Nkd1 and Axin established by a previous screen to identify Wnt inhibitors. We wondered whether endogenous Axin interacted with Nkd1 and Rnf25 in the same complex and immunoprecipitated lysates from HA-Nkd1 transfected HEK293T with either C2b for Axin or control antibodies. Both Rnf25 and HA-Nkd1 were co-precipitated with C2b antibodies but were absent from control precipitates. Elutes were then subjected to a second IP with anti-HA or control antibodies. The same observation was repeated under the knocking down condition of Dvl2 (Figure S10–S12). All of biochemical results revealed both Rnf25 and Nkd1 were co-precipitated with anti-HA but not control antibodies (Figure 2E), which suggested that Rnf25, Nkd1, and Axin formed a ternary complex.

### Rnf25 positively regulates canonical wnt signaling in the early embryonic development of vertebrate and the EMT

**Figure 3:**
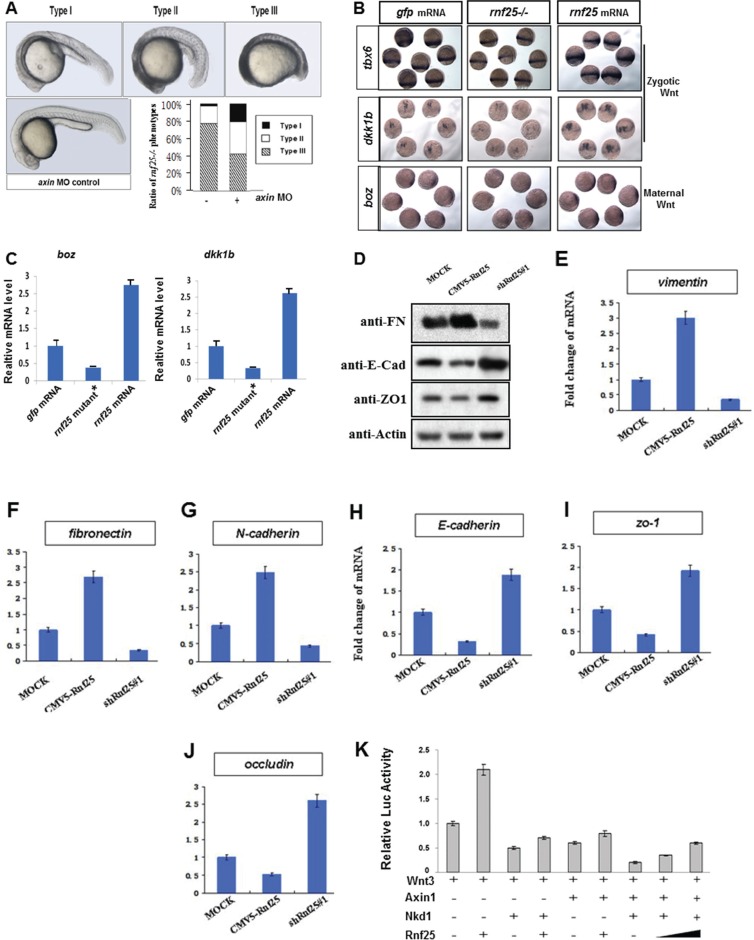
Rnf25 regulates zebrafish embryonic development and canonical Wnt signaling (**A**) Knockdown of *axin1* partially rescued the *rnf25* mutants in developing zebrafish morphology represented by the decreased ratio of Type III (the most severely impaired) *rnf25* mutants and the increased ratio of Type I (mildly impaired) *rnf25* mutants. The dose of axin morpholino injection or control morpholino was stabilized on 2 ng each embryo. (**B**) *rnf2*5 mutation (middle) or overexpression (right) injection affected zygotic and maternal Wnt signaling, represented by the *in situ* hybridization of zygotic Wnt targets *tbx6* and *dkk1b*, and maternal Wnt target *boz/dhama*. Embryos injected with the same amount of *gfp* mRNA (left) were served as loading-control. (**C**) The transcription of *boz/dhama* (left panel) and *dkk1b* (right panel) in zebrafish embryos were impaired by *rnf25* mutant and enhanced by *rnf25* mRNA injection. *gfp* mRNA injections were served as control. (**D**) Detecting the protein levels of E-Cadherin, Fibronectin and ZO-1 in MOCK, Rnf25 over-expression and Rnf25 knocking-down mK3 cells. (**E**–**G**) Fold change of transcripts of mesenchymal markers including *vimentin, n-cadherin* and *fibronectin* in mK3 cell system. (**H**–**J**) Fold change of transcript of epithelial markers *including zo-1, e-cadherin* and *occludin* in mK3 cell system. (**K**) Rnf25 over-expression restrained the inhibitory effect of Nkd1 and Axin on Wnt signaling in a dose dependent manner. HEK293T cells were transfected with indicated plasmids and treated with Wnt3 to initiate Wnt signaling. Axin and Nkd1 co-transfection significantly inhibited Lef1-Luc activity (lane7). The addition of Rnf25 decreased Lef1-Luc activity in a dose dependent manner (lane8, 9). Data obtained from triplicates are presented as the mean SD of a representative experiment. FN, Fibronectin; E-Cad, E-Cadherin.

In the early development of zebrafish, *axin1* genetic point mutant *mbl* (*masterblind*) could cause the eyeless phenotype. And we noticed that Rnf25 mRNA could partially phenocopy the *masterblind* mutants. Interestingly, the knockdown of *rnf25* in *mbl* heterozygous mutants rescues the eyeless phenotype in 30°C incubation (Figure S3). Furthermore, the zebrafish embryos that carry *rnf25* mutation generated by CRISPR/Cas9 strategy exhibited axis extension defects and malformed tail-fin derivatives, while injection of *axin* MO represented a rescue effect (Figure 3A). Whole mount *in situ* hybridization and real-time PCR were then carried out to investigate whether Rnf25 regulates Wnt target genes transcription (Figure 3B and 3C). During zebrafish early gastrulation, the maternal Wnt target *boz/dhama* was decreased in *rnf25* mutants. In contrast, increased expression of *boz/dhama* was displayed in embryos injected with *rnf25* mRNA (Figure 3B lower panel). Similarly, two zygotic Wnt targets in the mid-gastrulation stage, *dkk1b* and *tbx6*, were shown to be attenuated in *rnf25* mutants and enhanced by its overexpression (Figure 3B upper panels). Corresponding real-time PCR data for *boz/dhama* and *dkk1b* quantified the regulatory effect of Rnf25 on canonical Wnt signaling (Figure 3C).

Besides the early embryonic development, Mesenchymal-Epithelial Transition (MET) progress in the metanephridium was also regulated by Wnt signaling. The specific knock-down of Rnf25 by shRNA in mK3 cell have shown approximately a three-fold increase in the protein levels of E-Cadherin and ZO-1, two classical epithelial markers [17]; while the over-expression of Rnf25 inhibited their expression levels (Figure 3D). For the Fibronectin, a mesenchymal marker, above treatments have the opposite effects, respectively (Figure 3D). Next, by qPCR, we examined the mRNA level of the core EMT regulatory factors Vimentin, Fibronectin, N-Cadherin, ZO-1, E-Cadherin, and Occludin, and found that the mRNA expressions of Vimentin, Fibronectin and N-Cadherin were significantly down-regulated in shRNA-Rnf25 transfected mK3 cell (Figure 3E, 3F and 3G). Consistently, we observed the positive fold change of epithelial markers including ZO-1, E-Cadherin and Occludin in the same mK3 cell system (Figure 3H, 3I and 3J). On the other hand, the mRNA expressions of Vimentin, Fibronectin and N-Cadherin were upregulated in the Rnf25 overexpressed mK3 cell.

Nkd1-Axin interaction is particularly required for Nkd1 to antagonize Wnt signaling in zebrafish [18, 19], and the co-transfection of Nkd1 and Axin robustly decreased the protein level of Beta-Catenin in cultured mammalian cells (Figure S4). The effects of Rnf25, together with Axin and Nkd1, on the Wnt-induced Beta-Catenin mediated transcriptional activation were corroborated by Lef1-luciferase Reporter Assay in HEK293T cells. As was shown in Figure 3K, Lef1-luciferase reporter activity initiated by Wnt3 treatment (lane 1) was positively regulated by Rnf25 transfection (lane 2) and negatively influenced by Nkd1(lane 3) or Axin (lane 5) transfection. Co-transfection of Nkd1 and Axin seriously reduced Lef1-luciferase reporter activity (lane 7). The presence of Rnf25 slightly alleviated the inhibitory effect of Nkd1 (lane 4) or Axin (lane 6), while dose-dependently rescued the Lef1-luciferase reporter signal attenuated by Nkd1 and Axin co-transfection (lane 8, 9). These *in vivo* data indicated that Rnf25 is involved in early development of vertebrates and mesenchymal-epithelial transition processes via canonical Wnt signaling and supported that Rnf25 positively regulates canonical Wnt signaling by interacting with Nkd1 and Axin.

### Rnf25 disrupts Nkd1-Axin interaction independent of its E3 ubiquitin ligase activity

**Figure 4:**
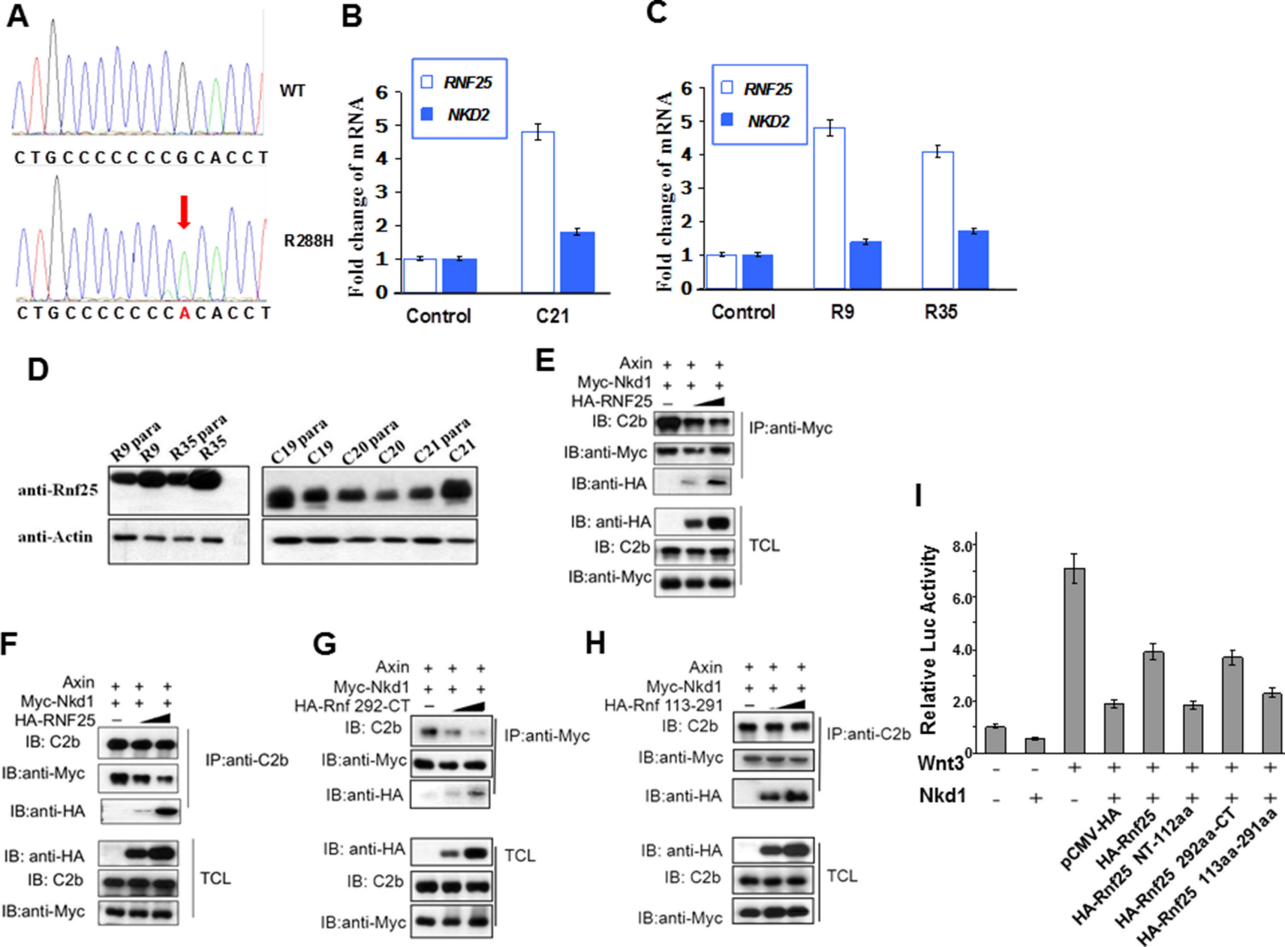
Rnf25 and Nkd1 maintain the balance of Wnt signaling *in vivo* (**A**) Sequencing results on human Rnf25 gene in tumor and para-carcinoma tissues. The red arrow indicated the same point mutation in three different tissues. (**B**) The relative transcriptional levels of *rnf25* and *nkd2* in carcinomatous and paracarcinomatous tissues from different colorectal tumors. (**C**) The relative transcriptional levels of *rnf25* and *nkd2* in carcinomatous and paracarcinomatous tissues from different renal tumors. (**D**) The protein level of Rnf25 in carcinomatous and paracarcinomatous tissues. R9 and R35 indicated Renal tumors, C19-C21 indicated Colorectal tumors. (**E**–**H**) HEK293T cells were transfected with Axin and Myc-Nkd1 together with 0.1-1ug HA-Rnf25 (E and F) or HA-Rnf25 292-CT (G) or HA-Rnf25 113–291 (H). Total cell lysates were immunoprecipitated with anti-Myc (E and G) or C2b (F and H) antibodies. Immunoprecipitates were detected by C2b, anti-HA and anti-Myc (upper panels) antibodies. (E) and (F) show that Rnf25 diluted Nkd1-Axin interaction. This inhibitory effect depends on the 292aa-CT Proline rich region of Rnf25 as was shown in (G), but not the 113aa-291aa RING domain of Rnf25 (H). Total cell lysates were western blotted using indicated antibodies and served as input control (lower panels). (**I**) Functional TOP-FLASH study on the functions of the Core-Peptides to the inhibitory role of Nkd1. Wnt3 group worked as the positive control, while the group with Wnt3-Nkd1 worked as the negative control. HA-tagged wild type and deletion mutants of Rnf25 were co-transfected as labeled in the figure.

To discover the functional relationship between Rnf25 and Nkd from clinical study, we sequenced the genomic DNA samples from 79 carcinomatous tissues using *nkd1*, *nkd2* and *rnf25* primers. Three R288H mutations in Nkd1 were detected in a colorectal tumor and 2 renal tumors (Lower panel in Figure 4A). In these three tumor tissues, the expression level of *rnf25* were elevated drastically more than that in paracarcinomatous tissues, while the expression level of *nkd2* were elevated moderately (C21, R9 and R35 in Figure 4B and 4C). The Rnf25 protein level in three tumor tissues also elevated than that in paracarcinomatous tissues, respectively (C21, R9 and R35 in Figure 4D). To gain insight into how Rnf25 interacts with Nkd1 and Axin, HEK293T cells were transfected with expression plasmids encoding Myc-Nkd1 and Axin, regardless of if a HA-Rnf25 gradient was introduced. In agreement with the Lef1-luciferase reporter assay results (Figure 3K), the binding of Nkd1 to Axin are specifically and dose-dependently diluted by Rnf25 as was unveiled by reciprocal IP and WB (Figure 4E and 4F). Moreover, consistent with the domain mapping of Rnf25 binding Axin (Figure 1E), the Rnf25 292aa-CT fragment and not the 113aa-291aa RING domain fragment, destabilized Nkd1-Axin interaction in the same condition (Figure 4G and 4H). Functionally, this “Core-Peptides” sequence involved in the full-length and the 292-CT of Rnf25 could restrain the inhibitory role of Nkd1 on TOP-FLASH readout (Figure 4I), whereas other two fragments only have negative effects. These findings from clinical samples and biochemical experiments suggested that Rnf25 disrupts the Nkd1-Axin interaction and activates canonical Wnt signaling independent from RING domain, which is responsible for its E3 ubiquitin ligase activity.

**Figure 5:**
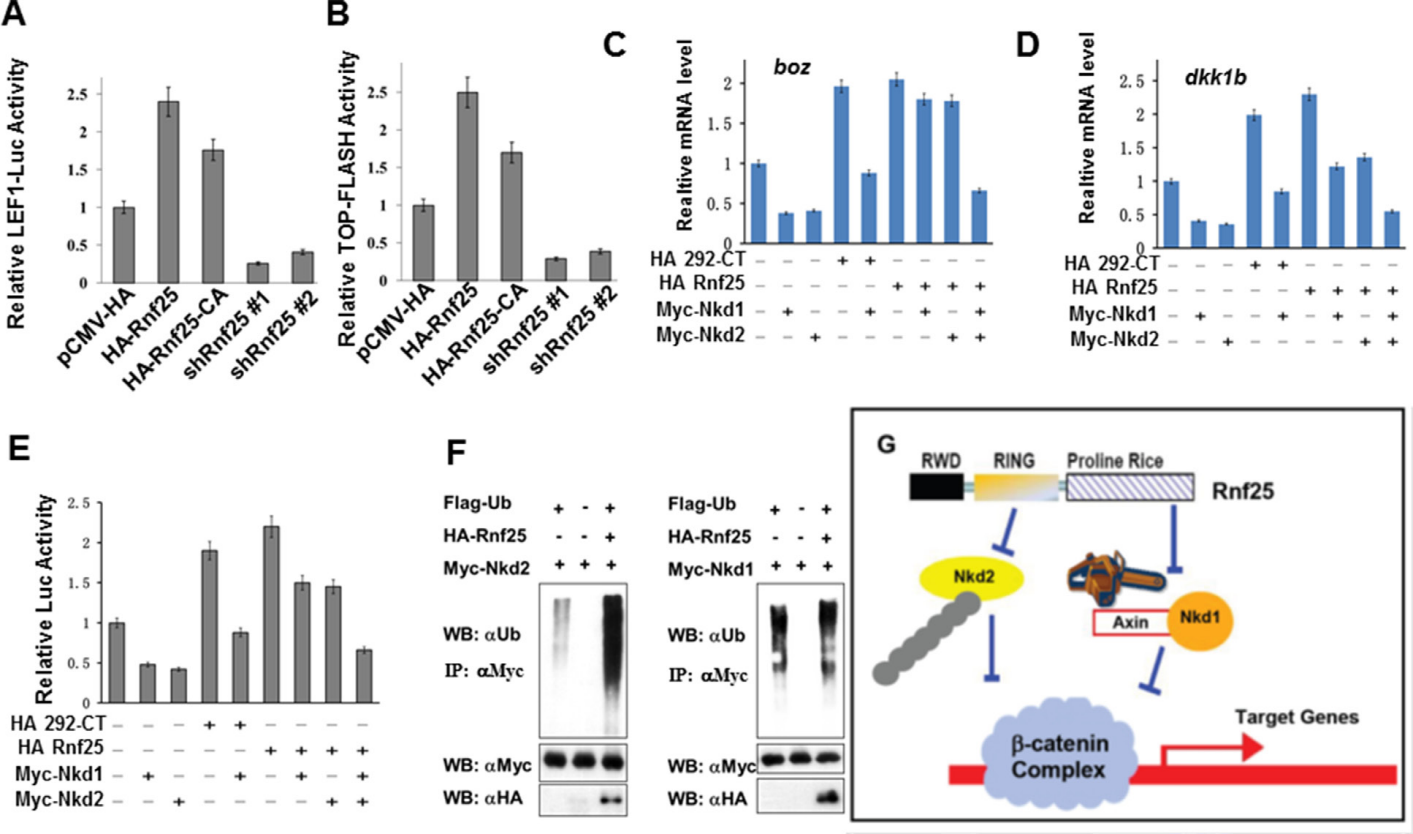
Rnf25 enhances Wnt signaling in an E3 ligase activity independent manner (**A** and **B**) In HEK293T cells, Rnf25 over-expression enhanced Lef1-Luc activity (panel A, lane2) and TOP-FLASH activity (panel B, lane2). The Rnf25 CA mutant, whose E3 activity was abolished, elevated Wnt signaling (lane3) compared with empty vector control (lane1). shRNAs against Rnf25 repressed Wnt signaling (lane 4 and 5). (**C**, **D** and **E**) The introducing of Nkd1 (lane5 in each panel) competently rescued the positive effects of Rnf25 292aa-CT on Wnt signaling. Co-transfection of Nkd1 and Nkd2 (lane9), but not Nkd1 (lane7) or Nkd2 (lane8) alone, rescued the Wnt signaling elevated by full length Rnf25 to the basal level indicated by qRT-PCR analysis of *boz/dhama* (C) and *dkk1b* (D). Expression in zebrafish embryos and Lef1-Luc reporter assay in cultured HEK293T cells (E). Data obtained from triplicates are presented as the mean SD of a representative experiment. (**F**) The ubiquitination assay of Rnf25. The overexpression system were immunoprecipitated by anti-Flag antibody (M2). The polyubiquitination of Nkd2 could be promoted by Rnf25 in left panel; however, Rnf25 has not effect on Nkd1's degradation in right panel (long exposing). (**G**) Working cartoon that Nkd1 and Nkd2 undergo different mechanisms for canonical Wnt signal regulation mediated by Rnf25/AO7.

To investigate the molecular mechanism of how Rnf25 promotes Wnt signaling, we employed an expression plasmid (Rnf25-CA) encoding Rnf25 RING domain mutant [14], whose E3 activity for Nkd2 substrate is crippled by introducing two point mutations at Cys-135 and Cys-138 in the conserved RING domain (Figure S5). HEK293T cells were transfected with wild type Rnf25, Rnf25-CA, and two independent shRNAs against to *rnf25*. Both Lef1-luciferase reporter assay and TOP-flash reporter assay showed that canonical Wnt signaling was activated by Rnf25 or Rnf25-CA, and blocked by *rnf25* shRNAs (Figure 5A and 5B). This was consistent with the Lef1-luciferase reporter assay indicating that either Rnf25 292aa-CT fragment or 113aa-291aa RING domain fragment promoted canonical Wnt signaling (Figure S6). The last observation raised an intriguing possibility that Rnf25 increased Wnt signaling via two independent manners, respectively mediated by the RING domain and the C-terminal fragment. Parallel experiments were conducted by measuring zebrafish Wnt activation in the context of over-expressed C-terminal Rnf25 or the full-length Rnf25. Real-time PCR assay suggested full-length and C-terminal fragment increase maternal and zygotic Wnt target genes (Figure 5C and 5D). Given that Rnf25 ubiquitylates Nkd2, the ubiquitination of Nkd1 is not mediated by Rnf25 (Figure 5F), our functional studies in culture cell and zebrafish embryos also revealed the association between Rnf25 C-terminal and Nkd1 in regulating Wnt signaling. Nkd1 over-expression greatly attenuated Wnt signaling, and this inhibitory effect could be significantly rescued by full-length Rnf25 or the C-terminal Proline-Rich fragment, while the N-terminal RWD or the RING domain fragment does not have such effect (Figure 5C, 5D and 5E).

These results proved that, when mediated by Rnf25, Nkd2 displayed significant degradation efficiency, whereas the level of Nkd1 ubiquitination stayed unaffected. Rnf25/AO7 displayed an E3-independent function by disrupting Nkd1-Axin interaction to positively regulate Wnt signaling both *in vitro* and *in vivo* (Figure 5G).

## DISCUSSION

First, we confirmed the direct interactions between Nkd1 and Axin1 and also between Nkd1 and Axin2/Conductin (Figure S7), in accordance with the idea that Axin2 functions similarly to Axin1. Nkd1 and Axin coordinately inhibit endogenous Wnt signaling while Nkd2 and Axin presented a much weaker association, which supported the hypothesis that Nkd1 is the Nkd orthologue and appears more specific for Wnt signaling. Be clearly different from Axin, the role of Axin2/Conductin for Wnt signaling varies to its cytoplasmic localization [20, 21]. Especially, Stephen Weiss and colleague reported that Axin2 could work as oncogene for colon carcinogenesis [22]. These clues and our unpublished observation that Conductin interacts less efficiently with Nkd1 than Axin with Nkd1 provided the point of interest about the Axin-Conductin functional difference in Nkd-Rnf25 pathway for further study.

In zebrafish embryonic development, the expression of *nkd2* is maternal and ubiquitous while *nkd1* activation is zygotic and substantially mirroring Wnt signaling [8]. In this context, a proposed model would be that Wnt signaling activation triggers the Wnt/Beta-Catenin pathway targeted gene transcription, and Nkds would become activated upon further Wnt activity. This activation of Nkds requires co-factors or at least binding partners to effectively inhibit further signaling of the Wnt pathway. Besides the previously reported Dvl, Axin and Beta-Catenin, other binding factors may also engage in the inhibitory effect of Nkds on Wnt signaling. We have shown here that Rnf25 binds Nkd1 and Nkd2 with different structural regions. In Rnf25, this interaction with Nkd2 is mediated by the RING domain (aa 113–291), a domain that is essential for its E3 ubiquitin ligase activity, leading to Nkd2 polyubiquitination and degradation [15]. The interaction with Nkd1 depends on the C-terminal Proline rich region (aa 292–513) of Rnf25 and is free from its E3 activity. We further demonstrated that Rnf25 disrupts the Nkd1-Axin interaction and therefore positively regulates Wnt signaling in both mammalian cell culture and zebrafish embryos. As Rnf25 may directly attenuate endogenous Nkd2 level by its E3 ubiquitin ligase activity, our data suggested that Rnf25 interacts with Nkd1 with an alternative functional domain and promotes Wnt signaling by disrupting Nkd1-Axin stability.

We have also verified the Nkd1-Axin, Rnf25-Nkd1 and Rnf25-Axin interaction via co-IP and GST pull-down, and postulated the existence of an Rnf25-Axin-Nkd1 ternary complex by two-step co-IP. As previous reviewed, Axin has been well established as a scaffold protein for multiple signaling pathways by a broad range of mechanisms [23]. The identification of Rnf25 as a novel Axin-interactor strengthened the idea that Axin is a key scaffold supporting multiple components in JNK, p53, TGFb, and Wnt. We determined that the C-terminal Proline rich region (aa 292–513) of Rnf25 is necessary and sufficient for its interaction with Axin, consistent to the yeast two hybridyzation result by RGS domain of Axin as bait. We also suggest an epistasis role of Axin on Rnf25 in Wnt signaling based on the observation that co-knockdown of *axin* not only rescued the phenotypes observed in *rnf25* mutants, but also re-enhanced Wnt targets expression level.

Despite the overwhelming evidence that Drosophila Nkd and its two vertebrate orthologs, Nkd1 and Nkd2, antagonize the Wnt/Beta-Catenin pathway via targeting the Dvl [24, 25], the observation that Nkd mutants preserving Dvl binding capability were ineffective in blocking transcriptions of Wnt target genes suggested that the mechanism by which Nkd inhibits Wnt is largely unknown [4, 18]. Subsequent studies demonstrated that Nkd2 regulates Dvl1 stability in a myristoylation-dependent manner [26], while Nkd1 binding to Dvl2 is independent of myristoylation [9]. In our supporting materials, although Dvl2 is co-precipitated with overexpressed Rnf25 in HEK293T cell, we only got negative result in GST-Pulldown experiment to test interaction between Dvl2 fragments and Rnf25 expressed in E. coli. It is possible that Rnf25 not interacts with Dvl2 directly, but could get together via scaffold proteins, for instance, Axin or Nkd1/2 (Figure S8 and S9). Moreover, the formation and function of Rnf25-Nkd-Axin complex were unaffected under knocking down condition of Dvl2 (Figure S10–S12).

In conclusion, we showed that Rnf25, in addition to leading the ubiquitination and proteasome-mediated degradation of Nkd2, specifically binds Axin and Nkd1 in an E3 ubiquitin ligase activity-independent manner, and thereby disrupts the Nkd1-Axin complex. Together with the Wnt signaling monitoring both mammalian cells and zebrafish embryos, Rnf25 reinforces Wnt signaling via Nkd1or Nkd2 by the different functional domains of itself and might serve as a molecular device facilitating Ndk1 and Nkd2 similar antagonizing function in Wnt signaling. The practical diagnostic methods and products based on Nkd1, Nkd2, and Rnf25 will be developed and applied in the immediate future.

## MATERIALS AND METHODS

### Micro-manipulation of zebrafish embryo

To knockdown axin1 genes function, Morpholino oligonucleotides were synthesized by Gene Tools company (A1-MO1: 5′- CATAGTGTCCCTGCACTCTGTCCCA-3′, refer to Steven Fong 2005 Development). All of oligo were dissolved in nuclease-free water to make a 20 μg/μL storage concentration. To cleaves double stranded DNA of zebrafish *rnf25*, a specific pair of primers for guide RNA (gRNA) was synthesized (5′- TAGGAGCTGAAGGACCGTGAG -3′ and 5′- AAACCTCACGGTCCTTCAGCT -3′ targeting 521–540 bp in NM_201183.1) for the CRISOR/Cas9 system (Clustered regularly interspaced short palindromic repeats). The mRNAs and morpholino oligonucleotides were injected into yolk of fertilized eggs at single-cell stage. For the *in vivo* reporter assay, reporter plasmid was co-injected with MO or mRNA at single-cell stage, and embryos were digested by passive lysis buffer at 90% epiboly to bud stage. All of DNA isolation and genotyping assay were performed after ISH staining.

### Yeast two-hybrid screen and construction of zebrafish rnf25 plasmids

A yeast two-hybrid screen using the RGS fragment of mouse Axin as bait was performed as described [8], and in this study, mouse Rnf25 was characterized. Zebrafish *rnf25, nkd1* and *nkd2* cDNA was isolated from an embryonic cDNA library by RT-PCR and subcloned into pBluescript to make antisense probe for *in situ* hybridization. The coding sequence of *rnf25* was cloned into vector pXT7 for mRNA synthesis.

### Preparation of rabbit polyclonal antibody against Rnf25

The DNA fragment encoding C-terminal human Rnf25 was inserted into pGEX4T and transformed into E. coli BL21 bacterial cells. The GST-Rnf25 fusion protein was induced with 1 mM IPTG and purified using glutathione beads. Rabbits were immunized with the purified GST-Rnf25 fusion protein (500 μg each in the complete Freund's adjuvant, Sigma) followed by three repetitions of boosting (250 μg each in the incomplete Freund′s adjuvant, Sigma). The Rnf25 antibody was then purified with the C-terminal human Rnf25 protein using an affinity purification method as previously described (24).

### Transient transfection, immunofluorescence, and coimmunoprecipitation

HEK293T cells were maintained in DMEM medium supplemented with 10% fetal bovine serum, 100 IU penicillin, 100 μg/ml streptomycin, and 2 mM glutamine. Transfections were performed in 60 mm dishes using Lipofectmine according to the manufacturer's instructions (Invitrogen). Cells were harvested at 36 hr posttransfection and lysed in a lysis buffer (20 mM Tris-HCl [pH 7.4], 150 mM NaCl, 1 mM EDTA, 1 mM EGTA, 1% Triton, 2.5 mM sodium pyrophosphate, 1 mMβ-glycerolphosphate, 1 mM sodium orthovanadate, 1 μg/ml leupeptin, 1 mM phenylmethylsulfonyl fluoride). Transiently transfected HEK293T cells in 60 mm dishes were lysed in a lysis buffer, sonicated three times for 5 s each, and centrifuged at 13,200 rpm for 30 min at 4°C. HA-tagged or Myc-tagged proteins were immunoprecipitated from the cell lysate with anti-HA (F–7), anti-Myc (9E10), or anti-Flag (M2, Sigma-Aldrich, Inc.) antibodies and Protein A/G Plus-agarose beads (Santa Cruz) as indicated. Immunoprecipitates or TCLs were analyzed bywestern blotting on Immobilon-P membranes (Millipore). After blocking with 5% nonfat milk in Tris-buffered saline with 0.1% Tween 20 for 1 hr, the membranes were probed with anti-HA, anti-Myc (9E10), anti-Ubiquitin (Merck, P4D1-A11), anti-Fibronectin (Abcam, ab2413), anti-E-Cadherin (Abcam, ab1416), anti-ZO1 (Abcam, ab59720), anti-BetaCatenin (Immunoway, YT0674), anti-Actin (Sungene, KM9001), or anti-FLAG M2 (Sigma) antibodies. Bound antibodies were visualized by enhanced chemiluminescence (Amersham Pharmacia) using peroxidase-conjugated antibodies (Sangon, AB10058, DAH012, DAH008; BBI, DAF1011, DAF1001, DAF1013).

The detail of fish maintain, plasmids construction, reverse transcription, *in vitro* transcription, reporter assay and the primers information were list in the supporting materials.

## SUPPLEMENTARY MATERIALS FIGURES AND TABLES


