# Supplementary material for: Rnf25/AO7 positively regulates wnt signaling via disrupting Nkd1-Axin inhibitory complex independent of its ubiquitin ligase activity
Source: Oncotarget. 2016 Mar 16;7(17):23850–9. doi: 10.18632/oncotarget.8126 (PMC5029668; doi:10.18632/oncotarget.8126)
Supplement: Supplementary file 1 [file oncotarget-07-23850-s001.pdf]

Rnf25/AO7 positively regulates wnt signaling via disrupting Nkd1-Axin inhibitory complex independent of its ubiquitin ligase activity

Supplementary Materials

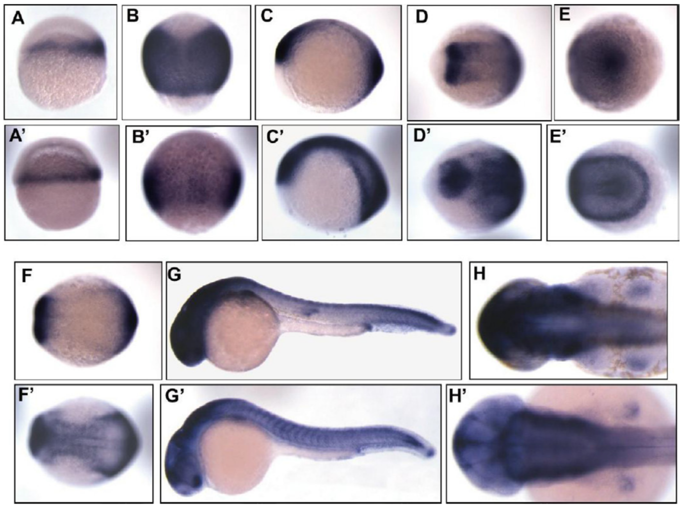

Supplementary Figure S1: *nkd1* and *axin2* shared similar expression patterns in Zebrafish embryo. (**A–H**) the spatio-temporal expression profiles of *axin*, (A'–H')the spatio-temporal expression profiles of *nkd1*.

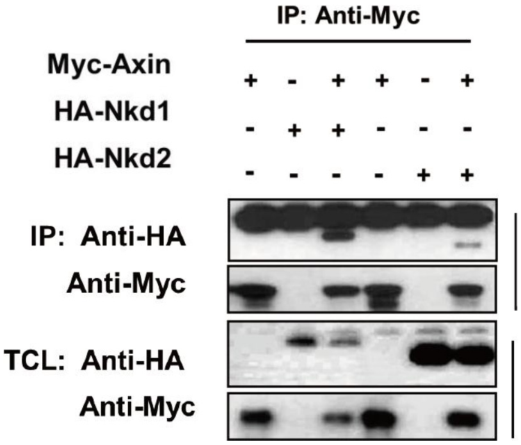

Supplementary Figure S2: Nkd1 and Nkd2 displayed different affinity to Axin. The Axin exhibited stronger interaction with Nkd1 (lane 3) than that with Nkd2 (lane 6) as was suggested by the IP results. All of cell lysates were incubated with anti-Myc antibody for 2 hours.

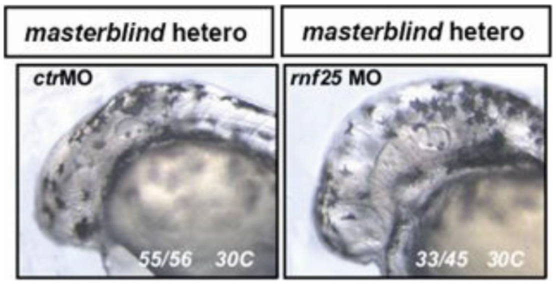

Supplementary Figure S3: The *masterblind* (*mbl*) zebrafish line develops small eye or eyeless phenotype under various incubation temperatures, due to mutation in the *axin* gene locus. The knockdown of *rnf25* in *mbl* heterozygous mutants rescues the eyeless phenotype in 30°C incubation.

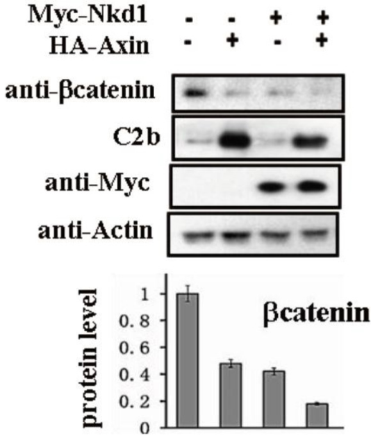

Supplementary Figure S4: Axin and Nkd1 co-transfection down regulates beta-catenin in HEK293T cells. The protein levels were detected by C2b, anti-βcatenin, anti-Myc and anti-Actin antibodies. Relative bands density of proteins were calculated by ImageJ software and listed at the bottom of the panel.

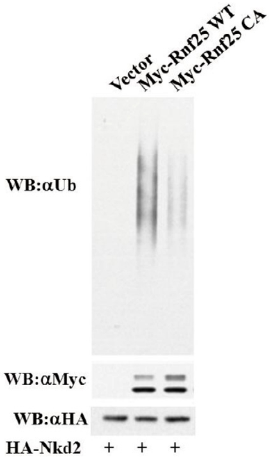

Supplementary Figure S5: By introducing two point mutations at Cys-135 and Cys-138 in the conserved RING domain of Rnf25, the Rnf25-CA mutant suggested defective ubiquitylation activity.

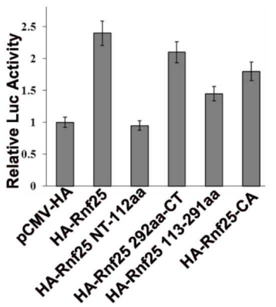

Supplementary Figure S6: The transfection of either full length Rnf25 (lane 2), the 113aa-291aa RING domain fragment (lane 4) or the 292aa-CT fragment (lane 5), but not the NT-112a fragment (lane 3) up-regulates Wnt signaling, compared with empty vector control (lane 1).

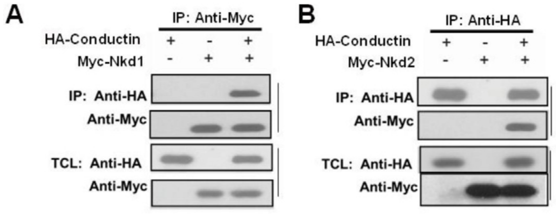

Supplementary Figure S7: Nkd1 and Nkd2 interacted with Axin2/Conductin in the over-expression system. The transfection dose of Myc-Nkd1 was 0.5 μg in panel A, the transfection dose of Myc-Nkd2 was 1.5 μg in panel B.

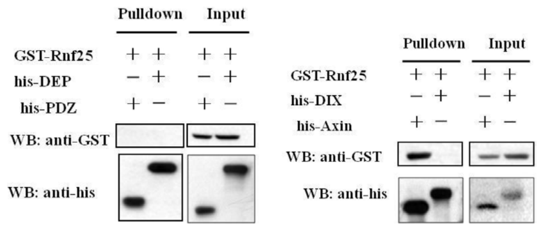

Supplementary Figure S8: *In vitro* GST-Pulldown experiment to test interaction between Dvl2 fragments and GST-Rnf25 expressed in E. coli. Left panel shown the interaction between Rnf25 with DEP or PDZ domains. Right panel shown the interaction between Rnf25 with DIX domain. The RGS domain of Axin was applied as positive control.

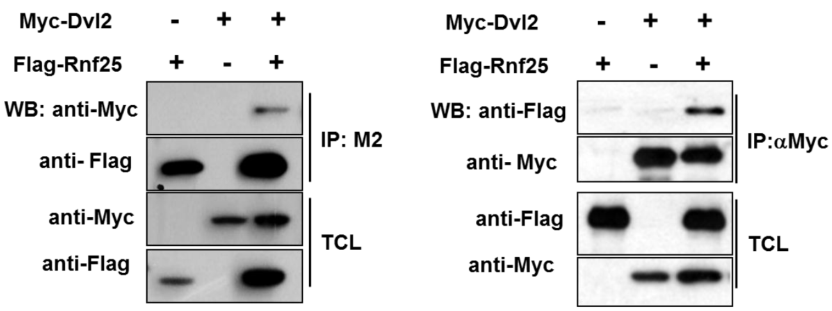

Supplementary Figure S9: Rnf25 and Dvl2 interaction in HEK293FT over-expression system.

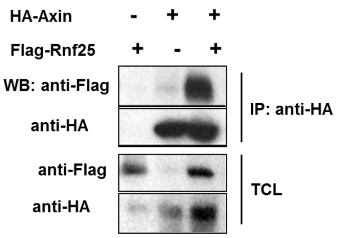

Supplementary Figure S10: The Rnf25-Axin interaction under knocking down condition of Dvl2.

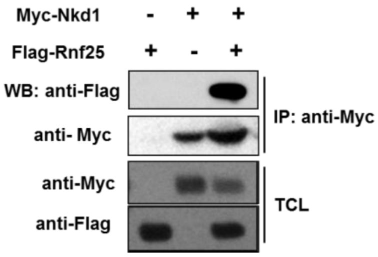

Supplementary Figure S11: The Rnf25-Nkd1 interaction under knocking down condition of Dvl2.

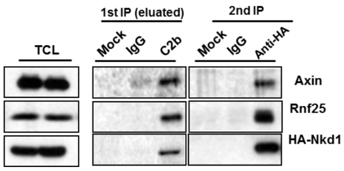

Supplementary Figure S12: The Axin-Nkd1-Rnf25 interaction under knocking down condition of Dvl2.

MATERIALS AND METHODS

Fish maintain

Zebrafish (*Danio rerio*), AB strain, were kept at 28.5°C under a light and dark cycle of 14 and 10 hours, respectively. Embryo stages are given in Hours Post Fertilization at standard temperature (hpf).

Plasmids construction

cDNA were ligated to pCMV5 vector by LIC method, some mRNA templates were subcloned to pxT7. All PCR products were confirmed by sequencing alignments. Human Rnf25 shRNAs were manipulated by pSico system using two pairs of primers with following sequences:

siF1:5′- Tcagatcaccatgggagatc TTCAAGA GAgatc tcccatggtgatctgTTTTTTC-3′

siR1:5′- TCGAGAAAAAAcagatcaccatgggagatc TCTCTTGAAgatctcccatggtgatctgA-3′

siF2:5′- Tattctcacggataacaaca TTCAAGAGA tgttg ttatccgtgagaatTTTTTTC -3′

siR2:5′- TCGAGAAAAAAattctcacggataacaaca TCT CTTGAAtgttgttatccgtgagaatA -3′

Reverse transcription and reporter assay

Total RNA was isolated from embryos at different stages using Trizol reagent (GIBCO). The first strand cDNAs synthesized from total RNAs were used as templates following the SuperScript Kit (Invitrogen). PCR reactions were performed for 30 cycles and a final extension for 6 min with internal *gapdh* control. HEK293T cells were transfected with 0.5 µg of pGL3-fos-7LEF-luciferase (provided by L. Williams), 0.1 µg of pCMV-β-galactosidase, 1 µg of β-catenin, and 1.5 µg of vector or of each of several constructs as indicated. Luciferase activities were measured as described previously (Xiong *et al.*,

2006). Data were presented as means from three separate experiments performed in triplicate.

RNA synthesis, whole-mount *in situ* hybridization

Capped mRNAs were synthesized using T7 Cap Scribe (Roche) according to the manufacturer's instructions. For preparation of digoxigenin labeled antisense probe, plasmid containing the *nkd1* cDNAs was linearized with *KpnI*. Linearized template DNA was transcribed in vitro with T7 polymerase using the digoxigenin-UTP (Roche). *In situ* hybridizations were performed as previously described.

Table1. Summary of primers information (Used in qRT-PCR and subcloning)

boz-F, 5′-CCGTAGCCGGTTGTGAAACAGC-3′
boz-R, 5′-TTCTTGAACCACACCCGCACAG-3′
tbx6-F, 5′- CAAGCTGGATTTGACTGCAA-3′
tbx6-R, 5′- GGGGTTTGTGAAGGCTGATA-3′
gapdh-F, 5′-GATACACGGAGCACCAGGTT-3′
gapdh-R, 5′-GCCATCAGGTCACATACACG-3′
dkk1b-F, 5′- GAAGAGTTCGTGTCCATCGC-3′
dkk1b-R, 5′- TAGTGTCTCTGGCATGTGTGC-3′
rnf25-F1, 5′- GCCATGGGCCATATGatggctgccgagagcgacgtc -3′
rnf25-R1, 5′- CCGGGATCCTCTAGAttctttggctctctctatgagc-3′
rnf25-F2, 5′- GCCATGGGCCATATGatcctgactgatagcaatattc-3′
rnf25-R2, 5′- CCGGGATCCTCTAGAgattgtgctagggaatctgg-3′
rnf25-F3, 5′- GCCATGGGCCATATGcaaacctctcaaaatcagcac-3′
rnf25-R3, 5′- CCGGGATCCTCTAGAtcagagcactcccctctttcc-3′
rnf25-F4, 5′-GCCATGGGCCATATGgaccagtgtcgtgacgtggacc-3′
rnf25-R4, 5′- CCGGGATCCTCTAGAgtcataaaggcagatgacacag-3′ for RFP-fusion 432 bp fragment ligase to DsRed fusion plasmid
zfRNF25-CA(C134S,C137S)-F catggaaacAgtgtcatcAgcctttatgactttaaggag
zfRNF25-CA(C134S,C137S)-R ctccttaaagtcataaaggcTgatgacacTgtttccatg
znkd1-F:5′- TTGCGGAAACATATGatgggtaaacttcattccaaac-3′
znkd1-R, 5′- CCGGGATCCTCTAGAtcaggactggtagaagtgatg-3′
znkd2-F: 5′- TTGCGGAAACATATGatggggaaacttcactccaaac-3′
znkd2-F: 5′- CCGGGATCCTCTAGAtcatgtctggtggtagtggtg-3′

shRNA against human Rnf25

siF1:5′- TcagatcaccatgggagatcTTCAAGAGAgatctcccatggtgatctgTTTTTTC-3′
siR1:5′- TCGAGAAAAAAcagatcaccatgggagatcTCTCTTGAAgatctcccatggtgatctgA-3′
siF2:5′- TattctcacggataacaacaTTCAAGAGAtgttgttatccgtgagaatTTTTTTC -3′
siR2:5′- TCGAGAAAAAAattctcacggataacaacaTCTCTTGAAtgttgttatccgtgagaatA -3′

In 36 colorectal tumors and 43 renal tumors, we detected 3 Nkd1 mutations. In these three tumor tissues, the expression level of *rnf25* were elevated dramatically, while the expression level of *nkd2* elevated moderately.

Supplementary Table S1: The clinical data sheet about the Nkd1-R288H mutation patients

Seq No.	Tissue	Sex	Age	Methodology	Ratio
C21	CRC	Male	48	SILS	4.78
R9	Renal	Male	46	nephrocentesis	4.82
R35	Renal	Female	51	nephrocentesis	4.05

Supplementary Table S2: The clinical data sheet about the Nkd1-WT patients (More than 3.0 fold change of rnf25 transcript)

Seq No.	Tissue	Sex	Age	Methodology	Ratio
R15	Renal	Male	53	nephrocentesis	3.88
R17	Renal	Female	47	nephrocentesis	3.61
H22	Hepatoma	Male	52	SILS	3.18
H29	Hepatoma	Male	57	SILS	4.15
H56	Hepatoma	Female	59	SILS	4.02
H61	Hepatoma	Female	49	SILS	3.54
H95	Hepatoma	Male	53	SILS	3.77
H103	Hepatoma	Male	52	SILS	3.41

In the revision stage, we utilized qPCR to detect *rnf25* level in hepatoma and breast carcinoma. Six samples were detected increased transcripts of *rnf25* in total 109 hepatoma tissues. However, no positive tissue was detected in all 122 breast carcinoma.
